# Supplementary material for: Activation and friction in enzymatic loop opening and closing dynamics
Source: Nat Commun. 2024 Mar 20;15:2490. doi: 10.1038/s41467-024-46723-9 (PMC10955111; doi:10.1038/s41467-024-46723-9)
Supplement: Supplementary file 1 — Supplementary Information [file 41467_2024_46723_MOESM1_ESM.pdf]

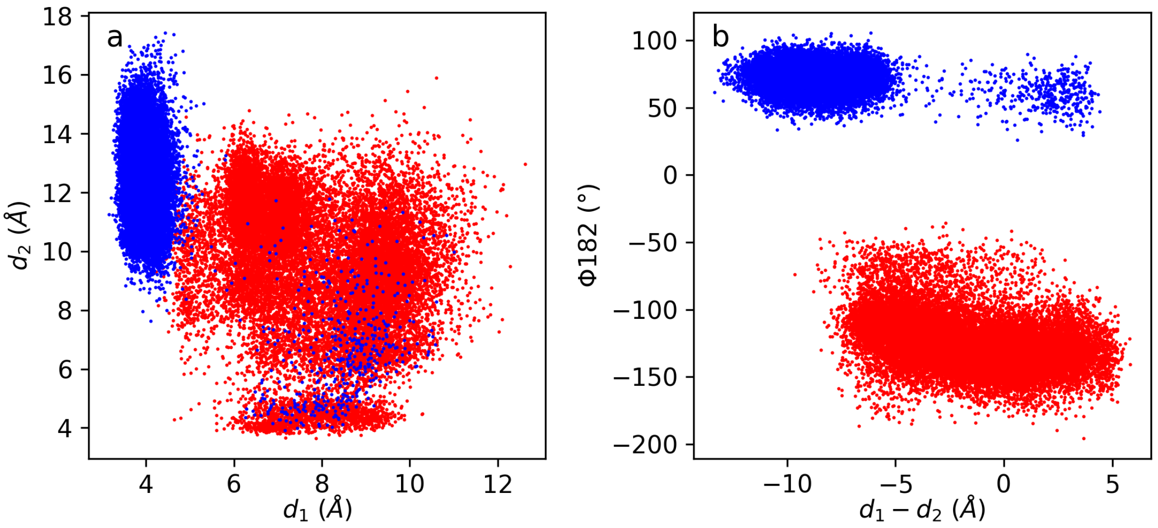

Supplementary Figure 1. Simulations of open and closed states in PTP1B. a) Distribution of values found for the distances d_1=(Asp181Cγ-Arg221Cζ) and d_2=(Asp181Cγ-Arg112Cζ) during open (red) and closed states (blue) simulations, showing that a function of these two distances cannot distinguish between the two states. b) Plot of the antisymmetric combination of distances (d1-d2) and the ϕ_{182} dihedral angle during the simulations of the two states, showing that the two closed and open conformations are clearly separated by the latter and not by a combination of the distances.

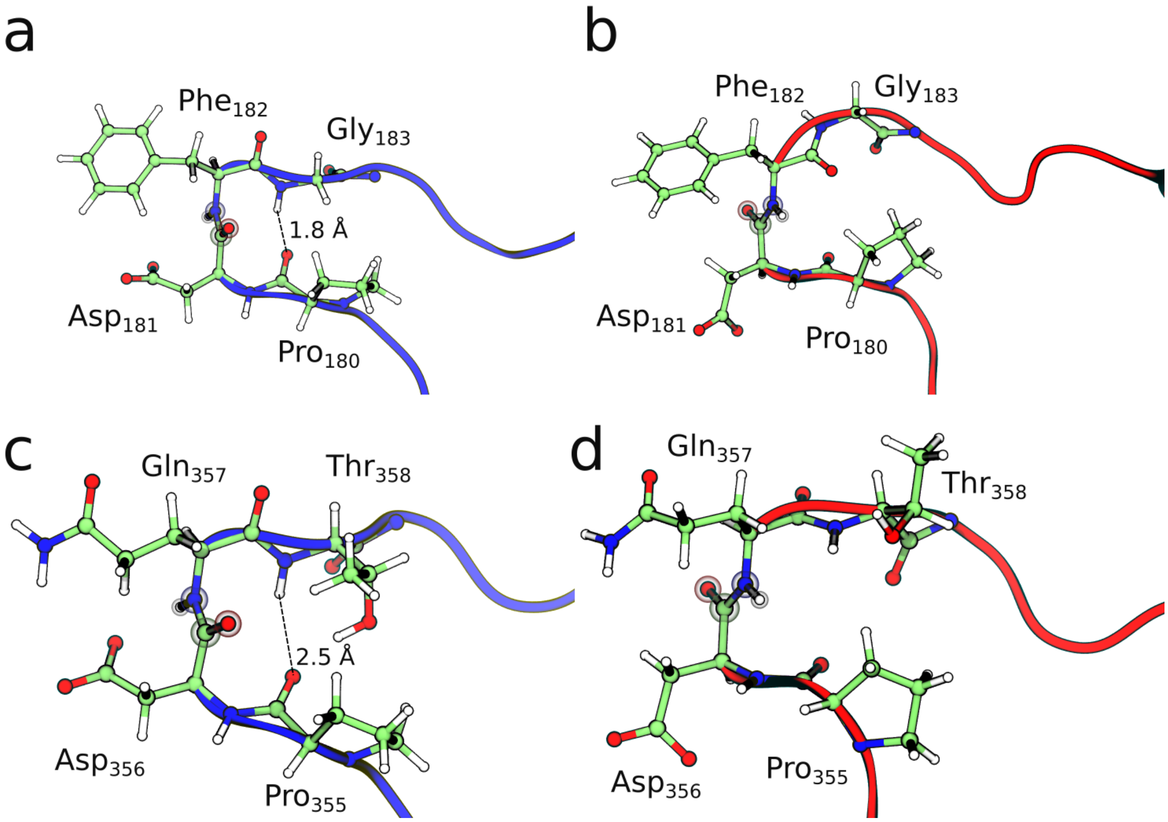

Supplementary Figure 2. β-turn in the WPD-loops of PTP1B (PDB structure 6B90) and YopH (PDB structures 1YPT and 2I42). a) β-turn formed by residues Pro180-Asp181-Phe182-Gly183 in the closed state of PTP1B, showing the hydrogen bond interaction between Gly183 and Pro180. b) same β-turn in the open state of PTP1B. c) β-turn formed by residues Pro355-Asp356-Gln357-Thr358 in the closed state of YopH, showing the hydrogen bond interaction between Thr358 and Pro355. d) same β-turn in the open state of YopH.

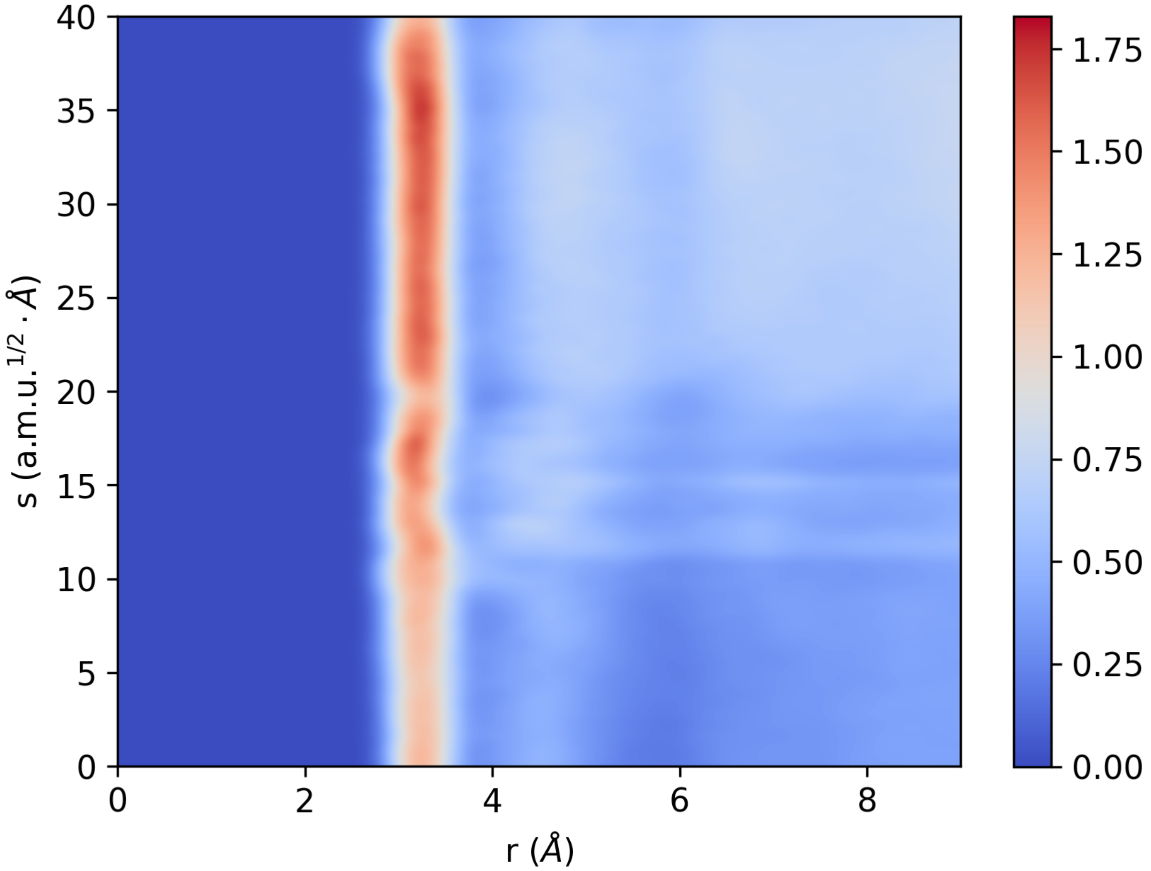

Supplementary Figure 3. Radial Distribution Function of water oxygen (Ow) atoms around the Asp181Cγ atom along the path-CV defining the transformation from the closed (small s) to the open conformation (large *s*). The x-axis is the Asp181Cγ-Ow distance and the value of the RDF is represented in the color scale on the right of the figure.

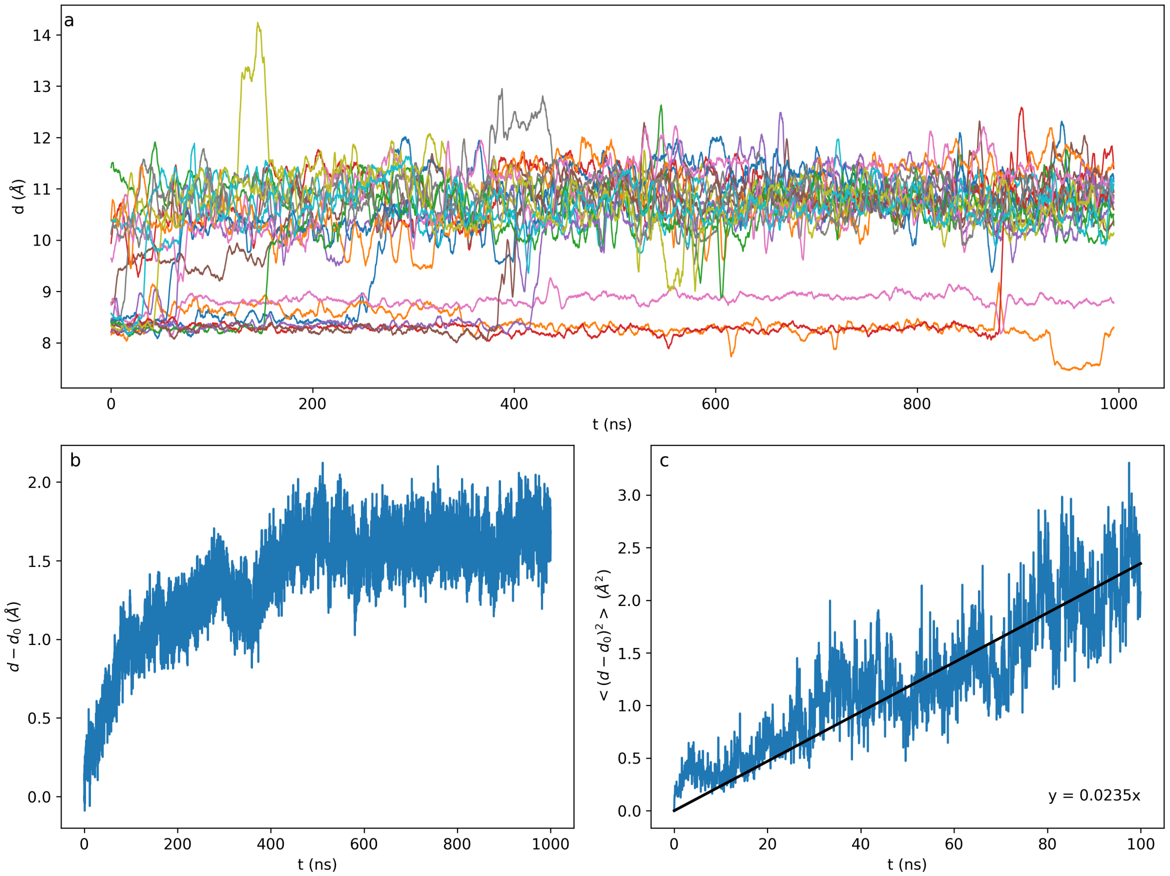

Supplementary Figure 4. Close to open transition of the WPD-loop in PTP1B after rotating the 181-182 peptide bond a) Evolution of the Asp181Cα-Gly220Cα distance (d) in 20 x 1 μs free MD trajectories started after the change in the ψ_{181}, ϕ_{182} torsional angles 18 out of the 20 trajectories resulted in a stable open loop. b) Averaged time evolution of the displacement in Asp181Cα-Gly220Cα distance with respect to the initial values. c) Linear time evolution of the average squared change in the Asp181Cα-Gly220Cα distance with the corresponding linear fit. The slope corresponds to twice the diffusion coefficient ($1.18 \cdot 10^{-2}$ Å$^2 \cdot$ns^{-1}).

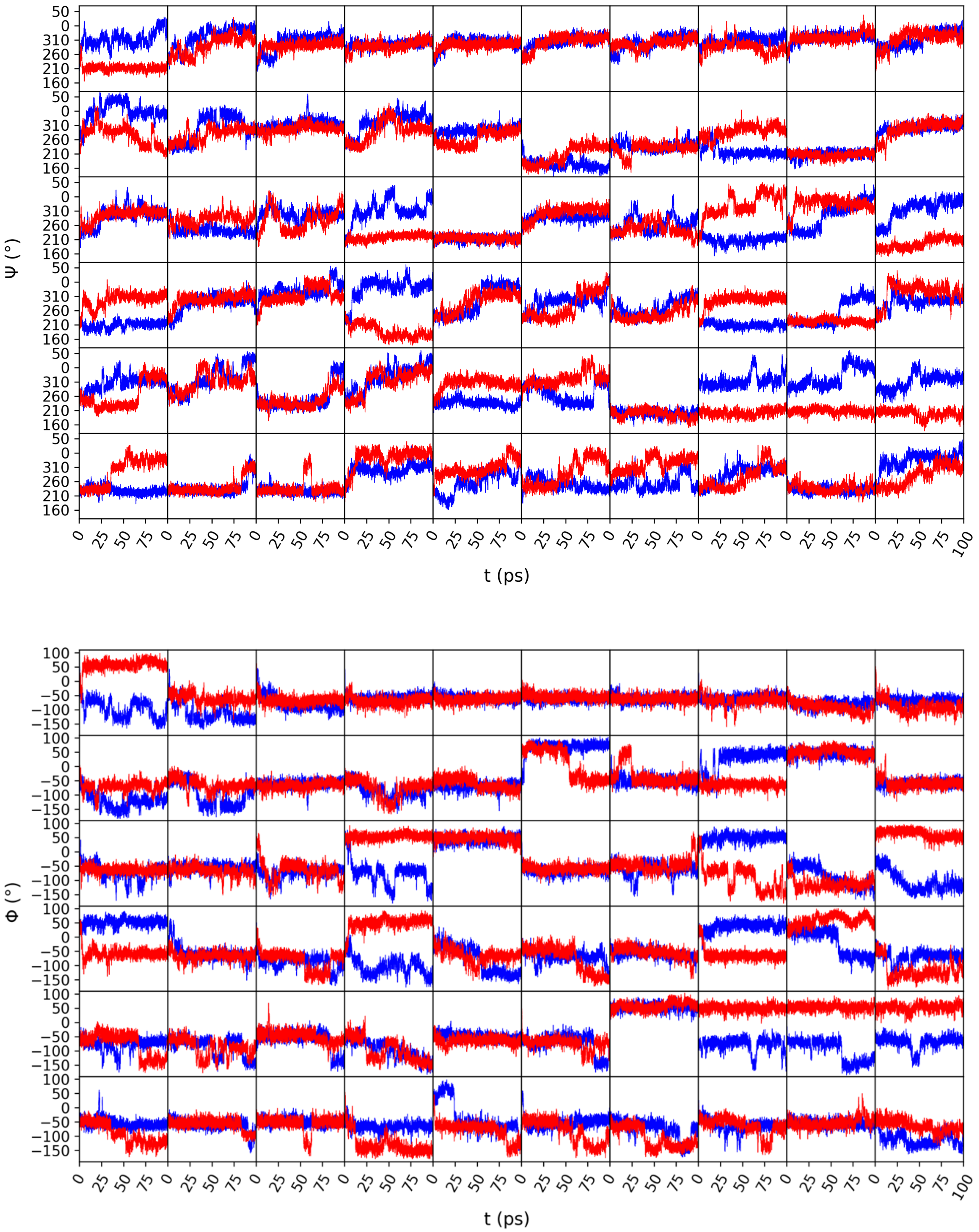

Supplementary Figure 5. Time evolution of the ψ_{181} (top panel) and ϕ_{182} (bottom panel) torsional angles for 60 free MD trajectories started from the TS located with the string method with random velocities selected from Maxwell-Boltzmann distribution. Blue and red colors correspond to trajectories propagated for positive (t>0) and negative (t<0) times until ±100ps. Details about rare event simulations are given in the Methods section.

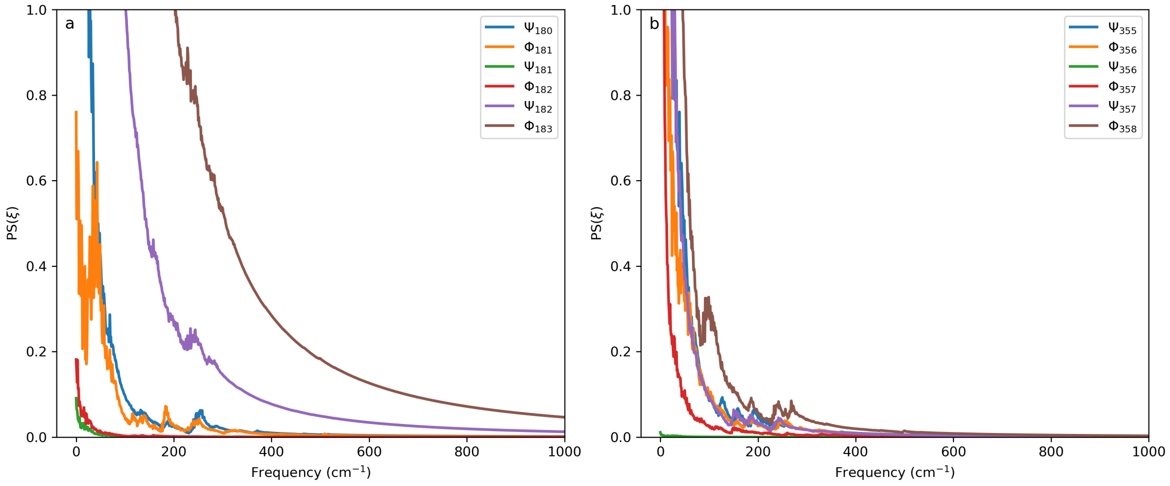

Supplementary Figure 6. Fourier transform of the time evolution of backbone torsions coupled to the reaction coordinate in **a)** PTP1B and **b)** YopH.

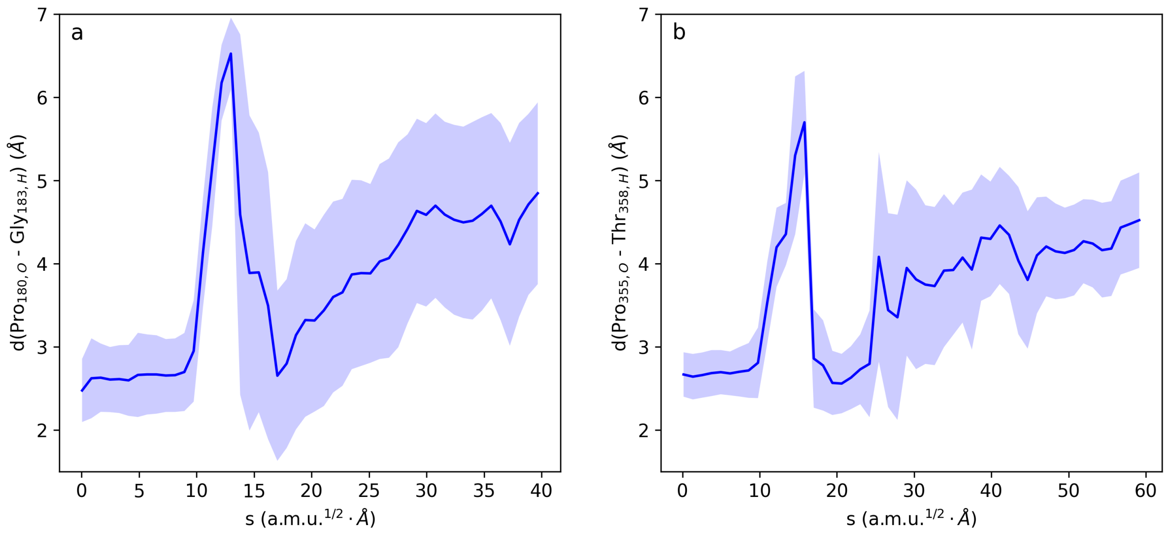

Supplementary Figure 7. Evolution of the hydrogen bond distance between the first and fourth residues of the β-turn of the WPD-loop along the path for the closed to open loop conformational change in **a)** PTP1B and **b)** YopH. The solid line corresponds to the average value and the shaded area to the confidence intervals (95% CI).

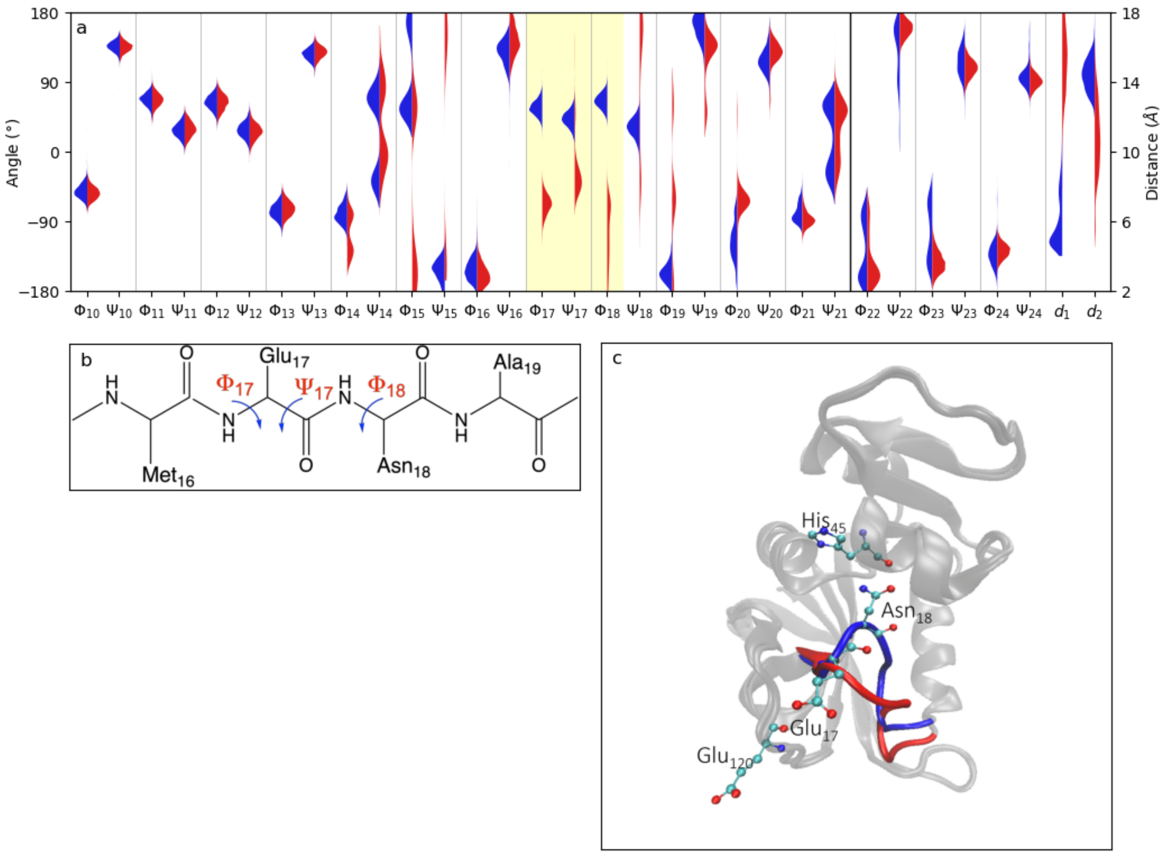

Supplementary Figure 8. a) Probability distributions of the backbone dihedral angles (ϕ and ψ) and selected distances (Asn18Cγ-His45Cα (d_1) and Asn18Cγ-Glu120Cα (d_2)) corresponding to the M20 loop of EcDHFR obtained from MD simulations of the open (red) and closed (blue) states; **b)** Definition of the torsional angles used for the string calculation of the closed to open transition of the M20 loop; **c)** Overlap of the x-ray structures of EcDHFR 1RX2 and 1RA1 corresponding to the closed (blue) and open (red) states for the M20 loop. The residues used to define the CVs employed in the string calculations are shown for the closed structure.

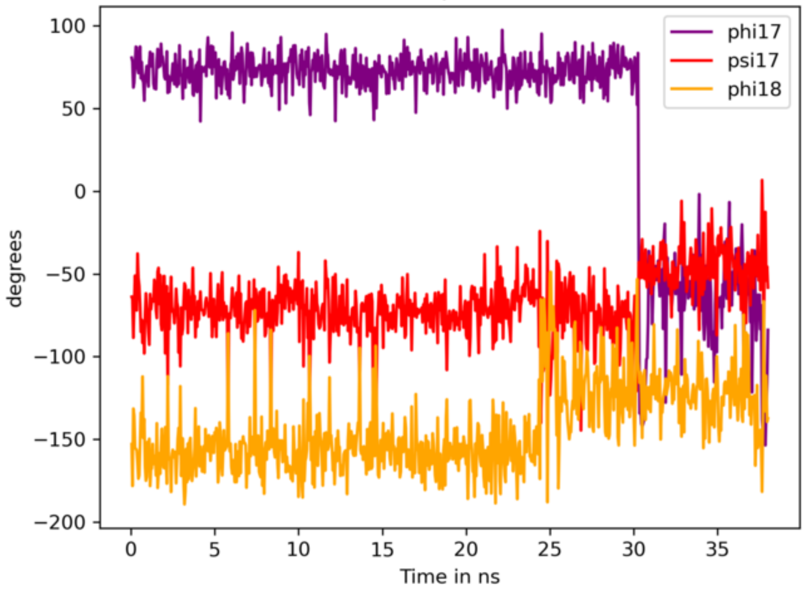

Supplementary Figure 9. Time evolution of the ϕ_{17}, ψ_{17}, ϕ_{18} torsional angles for a MD simulation started from the intermediate found in the free energy profile (see Figure 6a). The intermediate survives for some tens of ns before relaxing to the open state after a large change in ϕ_{17} accompanied with smaller changes in ψ_{17} and ϕ_{18}.

Supplementary Table 1. Details of ASM calculations for the conformational change of the WPD-loop in PTP1B and YopH enzymes.

	**PTP1B**	**YopH**	**EcDHFR**
String nodes	60	60	80
Number of CVs	4	5	5
REX period (fs)	500	500	500
String friction	1000	1000	1000
Force friction	50	50	50
Preparation (ps)	10	10	40
String optimization (ns)	30	10	8.5
Umbrella Sampling (ns)	15	10	20
Timestep (fs)	2	2	2

Supplementary Table 2. Collective variables (distances and torsional angles) employed in the ASM calculations of PTP1B, YopH and EcDHFR.

	**PTP1B**	**YopH**	**EcDHFR**
1	d($Asp_{181,C\gamma}$ – $Arg_{221,C\zeta}$)	d($Asp_{356,C\gamma}$ – $Arg_{409,C\zeta}$)	d($Asn_{18,C\gamma}$ – $His_{45,C\alpha}$)
2	d($Asp_{181,C\gamma}$ – $Arg_{112,C\zeta}$)	d($Asp_{356,C\gamma}$ – $Ser_{289,O\gamma}$)	d($Asn_{18,C\gamma}$ – $Glu_{120,O\gamma}$)
3	Ψ_{181}	d($Asp_{356,C\alpha}$ – $Gly_{408,C\alpha}$)	Ψ_{17}
4	Φ_{182}	Ψ_{356}	Φ_{18}
5	-	Φ_{357}	Φ_{17}

Supplementary Table 3. Initial guess for PTP1B ASM calculation.

**CV1 (Å)**	**CV2 (Å)**	**CV3 (°)**	**CV4 (°)**
3.5	14.0	130.0	80.0
6.0	9.0	-150.0	-50.0
9.0	4.0	-10.0	-150.0

Supplementary Table 4. Initial guess for YopH ASM calculation.

**CV1 (Å)**	**CV2 (Å)**	**CV3 (Å)**	**CV4 (°)**	**CV5 (°)**
3.5	12.5	6.5	140.0	75.0
7.0	8.0	9.0	240.0	-20.0
14.0	4.5	15.0	345.0	-105.0

Supplementary Table 5. Initial guess for EcDHFR ASM calculation.

CV1 (Å)	CV2 (Å)	CV3 (°)	CV4 (°)	CV5 (°)
4.5	14.6	48.9	62.2	75.0
10.6	11.8	11.3	159.0	-5.0
16.7	8.9	-26.2	256.0	-80.0

Supplementary Table 6. Main ingredients needed for the calculation of the rate constants for the closing/opening of the WPD-loop in PTP1B and YopH: average value of the modulus of the velocity along the reaction coordinate, activation free energies for the direct and inverse processes, initial friction (at t=0) expressed as a frequency, equilibrium and reaction frequencies along the reaction coordinate (obtained according to equations S2 to S4), Grote-Hynes and Kramers' evaluation of the transmission coefficient (equations S5 and S6).

Enzyme	$\langle\lvert\dot{s}\rvert\rangle_{\ddagger}$ ($amu^{1/2}\cdot Å\cdot fs^{-1}$)	$\Delta G^{\ddagger}_{s,open}$ ($kcal\cdot mol^{-1}$)	$\Delta G^{\ddagger}_{s,close}$ ($kcal\cdot mol^{-1}$)	$\frac{1}{2\pi c}\xi(0)^{1/2}$ (cm^{-1})	ω_{eq} (cm^{-1})	ω_{r} (cm^{-1})	κ	κ_{Kr}
PTP1B	0.0132	13.3	13.3	960	409	70	0.17	0.05
YoPH	0.0124	10.4	13.3	880	363	88	0.24	0.08

Supplementary Table 7. Summary Table of MD simulations

**Reliability and reproducibility checklist for molecular dynamics simulations**<br>***All boxes must be marked YES by acceptance unless "Response not needed if No".**	**Yes**	**No**	**Response**<br>**(Please state where this information can be found in the text)**
**1. Convergence of simulations and analysis**			
1a. Is an evaluation presented in the text to show that the property being measured has equilibrated in the simulations (*e.g.* time-course analysis)?	☒	☐	SI, Supplementary Methods
1b. Then, is it described in the text how simulations are split into equilibration and production runs and how much data were analyzed from production runs?	☒	☐	SI, Supplementary Methods
1c. Are there at least 3 simulations per simulation condition with statistical analysis?	☒	☐	SI, Supplementary Methods
1d. Is evidence provided in the text that the simulation results presented are independent of initial configuration?	☒	☐	SI, Supplementary Methods
**2. Connection to experiments**			
2a. Are calculations provided that can connect to experiments (*e.g.* loss or gain in function from mutagenesis, binding assays, NMR chemical shifts, J-couplings, SAXS curves, interaction distances or FRET distances, structure factors, diffusion coefficients, bulk modulus and other mechanical properties, *etc.*)?	☒	☐	Results section, '*Evaluation of the rate constant and the impact of friction in loop motion*' subsection
**3. Method choice**			
3a. Do simulations contain membranes, membrane proteins, intrinsically	☐	☒	

disordered proteins, glycans, nucleic acids, polymers, or cryptic ligand binding?			
3b. Is it described in the text whether the accuracy of the chosen model(s) is sufficient to address the question(s) under investigation (e.g. all-atom vs. coarse-grained models, fixed charge vs. polarizable force fields, implicit vs. explicit solvent or membrane, force field and water model, etc.)?	☐	☒	
3c. Is the timescale of the event(s) under investigation beyond the brute-force MD simulation timescale in this study that enhanced sampling methods are needed?	☒	☐	Methods section
If **YES**, are the parameters and convergence criteria for the enhanced sampling method clearly stated?	☒	☐	Methods section
If **NO,** is the evidence provided in the text?	☐	☒	
**4. Code and reproducibility**			
4a. Is a table provided describing the system setup that includes simulation box dimensions, total number of atoms, total number of water molecules, salt concentration, lipid composition (number of molecules and type)?	☐	☒	Input files provided in ttps://doi.org/10.5281/zenodo.10670397
4b. Is it described in the text what simulation and analysis software and which versions are used?	☒	☐	SI, Supplementary Methods
4c. Are other parameters for the system setup described in the text, such as protonation state, type of structural restraints if applied,	☒	☐	SI, Supplementary Methods

nonbonded cutoff, thermostat and barostat, etc.?			
4d. Are initial coordinate and simulation input files and a coordinate file of the final output provided as supplementary files or in a public repository?	☒	☐	See data availability section
4e. Is there custom code or custom force field parameters?	☒	☐	
If **YES**, are they provided as supplementary files or in a public repository?	☒	☐	ttps://doi.org/10.5281/zenodo.10670397

Supplementary Methods

Initial relaxation and free MD simulations

All the MD simulations were performed using *pmemd* program from Amber20 package.[1] The following protocol was used for the system relaxation in all cases (PTP1B, YopH and EcDHFR closed and open states):

1. 500 steps of steepest descent minimization followed by 500 steps of conjugate gradient. Harmonic restraints with force constant of 20 $kcal \cdot mol^{-1} \cdot Å^{-2}$ were applied to all solute atoms.
2. Gradual heating from 0 to 300 K during 200 ps of NVT MD. Here and in all subsequent simulations the timestep was set to 2 fs. The same harmonic restraints as in the minimization stage were kept.
3. 200 ps of NPT MD at 1 bar of pressure to relax the box size. The constraints of 20 $kcal \cdot mol^{-1} \cdot Å^{-2}$ were only applied to the backbone atoms.
4. 600 ps of NVT MD with backbone constraints of 20 $kcal \cdot mol^{-1} \cdot Å^{-2}$ for the first 200 ps, 4 $kcal \cdot mol^{-1} \cdot Å^{-2}$ for the next 200 ps and finally without the restraints for the last 200 ps.
5. 100 ns of unrestrained NVT MD for final relaxation.

The final structures from step 5 are available on GitHub (see Data availability).

10 x 100 ns unrestrained NVT MD simulations were then performed in both closed and open state to sample the conformational space accessible to both states. 10 snapshots taken every 5 ns from the last 50 ns of the trajectory obtained in step 5 were used as the initial structures for these simulations.

Forced conformational change

For PTP1B 20 simulations with forced conformational change of the β-turn in PTP1B were performed by starting with the structures taken after 50 and 100 ns of free NVT MD described above and first running 1 ns with 20 $kcal \cdot mol^{-1} \cdot rad^{-2}$ harmonic restraints to force the two dihedral angles to the values corresponding to the "open" conformation. Subsequently, for each of the 20 resulting structures 1 μs of unbiased NVT MD was performed.

Adaptive string method

The parameters of the adaptive string method (ASM) simulations are summarized in Supplementary Table 1.

For the detailed description of the parameters please refer to the original publication.[2] The statistical uncertainty of each PMFs was estimated dividing the sampling in five equally sized pieces and obtaining a PMF for each of them.

Supplementary Tables 2-5 provide the collective variables (CVs) employed for each enzyme and the initial guess used during the ASM simulations. Note that in all enzymes we used the torsional angles that define the rotation of a particular peptide bond, Asp181-Phe182 in PTP1B, Asp356-Gln357 in YopH and in EcDHFR. Regarding the displacement of the loop, we used two distances in the case of PTP1B, those defining the salt-bridge interactions establishes by the catalytic Asp181 in the closed state (with Arg221) and in the open state (with Arg112). In the case of YopH we also used the salt-bridge distance established in the closed state between Asp356 and Arg409. However, the interaction found in the open state for the catalytic Asp356 is a weaker hydrogen bond with the hydroxyl group of Ser289. For that reason, we also added a distance between the Cα atoms of Asp356 and Gly408 to correctly describe the opening of the loop. For EcDHFR we selected the torsional angles that define the rotation of the peptide bond Glu17-Asn18 and the distances Asn18Cγ-His45Cα and Asn18Cγ-Glu120Cα. The first distance corresponds to a close contact formed in the closed state, while the distance with respect to Glu120 defines the positioning of the Met20 loop relative to the FG one. These loops become close in the open state.

Grote-Hynes simulations

To analyze the friction acting on the reaction coordinate (*s*) and obtain the transmission coefficient with Grote-Hynes theory, two sets of 60 simulations were performed with the system restrained to the vicinity of the transition state (TS). The simulations were initiated from TS structures extracted from trajectories obtained during umbrella sampling simulations.

The first set of 60 x 25 ps NVT simulations was used to obtain the curvature of the potential of mean force (PMF) at the TS to derive the equilibrium frequency. To achieve this, a harmonic bias with a force constant of 200 kcal·mol^{-1} ·(a.m.u)$^{-1}$·Å^{-2} was applied, centered at the RC value corresponding to the TS (12.72 a.m.u.$^{1/2}$· Å for PTP1B and 15.70 a.m.u.$^{1/2}$· Å for YopH).

The second set of 60 x 20 ps simulations was used to obtain the friction acting on *s* and its power spectrum. To ensure the bias had a much higher frequency than the rest of the vibrations in the system, a much higher force constant of 10000 kcal·mol^{-1} ·(a.m.u)$^{-1}$·Å^{-2}was

employed. To ensure simulation stability, the timestep was reduced to 0.1 fs and structures were stored every 10 steps (this is, every 1 fs).

To decompose the friction, the topology files were modified to retain only the components of interest (solute, dihedrals, etc.). For each modified topology file, the total force was projected onto the reaction coordinate (*s*). The friction was then obtained as the autocorrelation of the projected force.

Supplementary Notes

Limiting behaviors in Grote Hynes Theory

Grote-Hynes (GH) theory[3] is based in a Generalized Langevin Equation (GLE) where a constant friction is substituted by a time-dependent friction kernel. Assuming mass-weighted coordinates, the equation for the motion along a reaction coordinate called s at the transition state (TS) region can be written as:

$$\mathrm{m} \cdot \ddot{s} = -\frac{\partial \mathrm{G}(s)}{\partial s} - \mathrm{m} \int_0^{\mathrm{t}} \xi(\mathrm{t}-\tau) \cdot \dot{s}(\tau) \cdot \mathrm{d}\tau + \mathrm{R}(\mathrm{t}) \qquad \text{(S1)}$$

On the right-hand side, the first term is the force arising from the underlying equilibrium free energy profile along the reaction coordinate $G(s)$. In the integral of the second term appears the time-dependent friction, or friction kernel $\xi(t-\tau)$, while the last term $R(t)$ is a zero-mean value fluctuating force arising from the coupling of all the remaining degrees of freedom with the reaction coordinate s.

The free energy profile is assumed to be an inverted parabola:

$$\mathrm{G}(s) = \mathrm{G}(s^{\ddagger}) - \frac{1}{2}\omega_{\mathrm{eq}}^2 \cdot (s - s^{\ddagger})^2 \qquad \text{(S2)}$$

The time dependent friction is the time correlation function of the fluctuating forces acting along the reaction coordinate: averaged friction kernel and the fluctuating forces are related by the autocorrelation of the forces:

$$\xi(\mathrm{t}) = \frac{1}{\mathrm{k_B T}} \langle \mathrm{R}(0) \cdot \mathrm{R}(\mathrm{t}) \rangle_{s^{\ddagger}} \qquad \text{(S3)}$$

The GLE can be also conveniently written in terms of frequencies:

$$\omega_{\mathrm{r}}^2 - \omega_{\mathrm{eq}}^2 + \omega_{\mathrm{r}} \cdot \int_0^{\mathrm{t}} \xi(\mathrm{t}) \cdot \mathrm{e}^{-\omega_{\mathrm{r}} \cdot \mathrm{t}} \cdot \mathrm{dt} = 0 \qquad \text{(S4)}$$

where ω_r is the frequency for crossing the barrier under the effect of the friction. The frequency is equal to the equilibrium frequency if friction is absent and smaller in other cases. Then, in GH theory the transmission coefficient can be evaluated as:

$$\kappa_{\mathrm{GH}} = \frac{\omega_{\mathrm{r}}}{\omega_{\mathrm{eq}}} \qquad \text{(S5)}$$

GH theory predicts several regimes for the impact of the friction on the motion along the reaction coordinate and then on the transmission coefficient, depending on the relative time scales of the motion along the reaction coordinate and the coupled environmental motions that give raise to the friction:[4]

1) *Adiabatic or Kramers' regime*:
When most of the time dependent friction is exerted during barrier crossing due to rapid solvent dynamics, then the frequency dependence of the friction can be ignored, and equation (S4) can be simplified to Kramers' equation:[5]

$$\omega_{Kr}^2 - \omega_{eq}^2 + \omega_{Kr} \cdot \int_0^t \xi(t) \cdot dt = 0 \quad \text{(S6)}$$

This regime requires rapid and complete relaxation of the environment during barrier crossing. In the context of chemical reactions, Kramers' regime leads to an overestimation of friction effects and then an underestimation of the transmission coefficient.[6]

2) Nonadiabatic or frozen environment regime:

In this case the time scale of the reaction is too short compared to motions of the environment. In this case the nonadiabatic reaction frequency is given by:

$$\omega_{na}^2 - \omega_{eq}^2 + \xi(t = 0) = 0 \quad \text{(S7)}$$

In this case the reaction frequency is reduced by the resistance or friction of a frozen environment that becomes a delta function centered at zero frequency.

3) Polarization or caging regime:

The dynamics of system off the TS responds to a free energy well rather than to a barrier because the lack of environmental motions traps the system in a cage. In this case environmental motions are essential to complete the process. This regime is identified when the zero-frequency friction overcomes the equilibration frequency and then the nonadiabatic frequency becomes imaginary:

$$\xi(t = 0) > \omega_{eq}^2 \quad \text{(S8)}$$

Complete equation (S4) must be used to calculate the reaction frequency and the transmission coefficient.

Supplementary References

1. Case, D. A. *et al.* AMBER 2020.
2. Zinovjev, K. & Tuñón, I. Adaptive Finite Temperature String Method in Collective Variables. *J. Phys. Chem. A* 121, 9764–9772 (2017).
3. Grote, R. F. & Hynes, J. T. The stable states picture of chemical reactions. II. Rate constants for condensed and gas phase reaction models. *J. Chem. Phys.* 73, 2715–2732 (1980).
4. Gertner, B. J., Wilson, K. R. & Hynes, J. T. Nonequilibrium solvation effects on reaction rates for model SN2 reactions in water. *J. Chem. Phys.* 90, 3537–3558 (1989).
5. Kramers, H. A. Brownian motion in a field of force and the diffusion model of chemical reactions. *Physica* 7, 284–304 (1940).
6. Roca, M., Moliner, V., Tuñón, I. & Hynes, J. T. Coupling between Protein and Reaction Dynamics in Enzymatic Processes: Application of Grote−Hynes Theory to Catechol O-Methyltransferase. *J. Am. Chem. Soc.* 128, 6186–6193 (2006).
