# Supplementary material for: Activation and friction in enzymatic loop opening and closing dynamics
Source: Nat Commun. 2024 Mar 20;15:2490. doi: 10.1038/s41467-024-46723-9 (PMC10955111; doi:10.1038/s41467-024-46723-9)
Supplement: Supplementary file 2 — Description of Additional Supplementary Files [file 41467_2024_46723_MOESM2_ESM.pdf]

Description of Additional Supplementary Files:

Supplementary Movie 1: This file contains a movie of the evolution of the WPD-loop in PTP1B along the Minimum Free Energy Path determined with the string method.
